# RETRACTION: Nrf3 Promotes 5‐FU Resistance in Colorectal Cancer Cells via the NF‐κB/BCL‐2 Signaling Pathway In Vitro and In Vivo

**DOI:** 10.1155/jo/9754059

**Published:** 2026-07-16

**Authors:** Journal of Oncology

RETRACTION: B.‐Q. Cai, W.‐M. Chen, J. Zhao, W. Hou, and J. C. Tang, “Nrf3 Promotes 5‐FU Resistance in Colorectal Cancer Cells via the NF‐κB/BCL‐2 Signaling Pathway In Vitro and In Vivo,” *Journal of Oncology*, 2021, 9355555, 12 pages, 2021, https://doi.org/10.1155/2021/9355555.

The above article, published online on 09 November 2021 in Wiley Online Library (https://wileyonlinelibrary.com), has been retracted by John Wiley & Sons Ltd.

The retraction has been agreed due to figure concerns initially raised on PubPeer [1] regarding an article by some of the same authors [2]. Specifically:

• In Figure 5f, the right‐most three western blot bands in the Nrf3 group under the SW620/shNrf3 blots appear to have been mirrored 180 degrees from Figure 4c in [2].

Additionally, upon further investigation by the publisher, other concerns were found, specifically:

• In Figure 6d, the panel for Bcl‐2 under the DMSO column in the HT29/Nrf3 group appears to have been duplicated from Figure 6c of [2], after being cropped and rotated 270 degrees.

Due to these concerns, the publisher no longer has confidence in the validity of the conclusions. Therefore, the article is retracted.

## References

[1] Tulipa Fosteriana, “12-Epi-Napelline Inhibits Leukemia Cell Proliferation via the PI3K/AKT Signaling Pathway In Vitro and In Vivo”, *PubPeer*, July 2021, https://pubpeer.com/publications/3AEC116E3CDFBEF054C2D03D9E85B3. 10.1155/2021/6687519 PMC8266464 34306152

[2] Han J. , Hou W. , Cai B. , Zhang F. , and Tang J. , 12-Epi-Napelline Inhibits Leukemia Cell Proliferation via the Pi3k/Akt Signaling Pathway In Vitro and In Vivo, Evidence-Based Complementary and Alternative Medicine. (2021) 1–12, 10.1155/2021/6687519.

